# Single nucleotide polymorphisms in *SAR1A* coding regions in sickle cell disease and their potential miRNA binding sites

**DOI:** 10.1002/jha2.542

**Published:** 2022-08-03

**Authors:** Chutima Kumkhaek, Christine Kim, Gulriz Kurban, Jianqiong Zhu, Wulin Aerbajinai, James G. Taylor, Griffin P. Rodgers

**Affiliations:** ^1^ Molecular and Clinical Hematology Branch National Heart, Lung and Blood Institute National Institutes of Health Bethesda Maryland USA; ^2^ Center for Sickle Cell Disease Howard University Washington District of Columbia USA; ^3^ Department of Medicine Howard University Washington District of Columbia USA

**Keywords:** *hydroxyurea*, *miRNA*, SAR1A, sickle cell disease, single nucleotide polymorphisms

Sickle cell disease (SCD) is the most common severe monogenetic disorder and displays marked phenotypic heterogeneity. The clinical diversity of SCD derives from the inter‐individual genetic background and other factors, including sociodemographic, socioeconomic, or environmental conditions [1]. For example, the variation of fetal hemoglobin (HbF) levels in SCD patients is influenced by the genetic loci inside or outside the β‐globin gene cluster, such as single nucleotide polymorphisms (SNPs) in the quantitative trait loci *XmnI‐HBG2, BCL11A*, and *HBS1L‐MYB* [2]. Hydroxyurea or hydroxycarbamide (HU) is a major therapeutic option for homozygous SCD and is well established as an agent for inducing HbF levels [3, 4]. An increase in the levels of HbF lessens the clinical severity of SCD. However, the increase in HbF in response to HU therapy varies considerably among SCD patients [5].

We have previously reported that SNPs in the secretion‐associated RAS‐related GTPase 1A (*SAR1A*) gene promoter contribute to interindividual differences in regulation of HbF expression, as well as response to HU, in SCD patients [6]. SAR1A is a member of the small GTPase protein family and regulates the formation or assembly of the endoplasmic reticulum (ER)‐derived coat protein complex II vesicles involved in the ER‐to‐Golgi apparatus export of proteins [7]. Interestingly, we found that *SAR1A* was a specific HU‐inducible gene and also demonstrated that HU‐induced *SAR1A* activates γ‐globin expression through the induction of GATA‐2 and the Giα/JNK/Jun pathway in human erythroid cells [8, 9]. Furthermore, the SNPs in the *SAR1A* promoter were found to be significantly associated with changes in HbF levels in both adults and children with SCD following HU treatment [6, 10]. Collectively, these observations suggest that *SAR1A* plays a pivotal role in HbF expression.

In this study, we hypothesized that SNPs in *SAR1A* coding regions affect HU‐induced HbF levels in SCD patients and determined the SNPs within the *SAR1A* 3ʹ untranslated region (UTR) as potential microRNA (miRNA) binding sites. Therefore, we expanded on our previous study [6] to investigate the association of SNPs in *SAR1A* coding regions with hematologic laboratory values in adults with SCD treated with HU. miRNAs are small noncoding RNAs that induce translational repression or decay, primarily by targeting the mRNA 3ʹ UTR [11]. Recently, Das et al. demonstrated that SNPs within miRNA binding sites can affect HbF expression [12]. miRNAs have also been shown to play an important role in HU‐mediated HbF induction, as well as to be related to the clinical heterogeneity of SCD [13, 14]. Furthermore, we examined SNPs within the *SAR1A* 3ʹ UTR as potential miRNA binding sites.

To identify SNPs in *SAR1A*‐coding regions, we sequenced all eight *SAR1A* exons in 32 adults with SCD undergoing HU therapy. Three (rs56090714, rs3812693, and rs56381518) and 15 (rs78341510, rs114346554, rs72807054, rs201493587, rs1470556171, rs2394643, rs80028936, rs7919647, rs115340990, rs15801, rs1046747, rs79535872, rs7653, rs1280408553, and rs10586) variants were identified in exons 1 and 8, respectively (Figure 1A). No SNPs were detected in exons 2–7. Among these, rs7919647 in exon 8 had the highest frequency (24.4%) in the cohort examined (Figure 1B).

**FIGURE 1:**
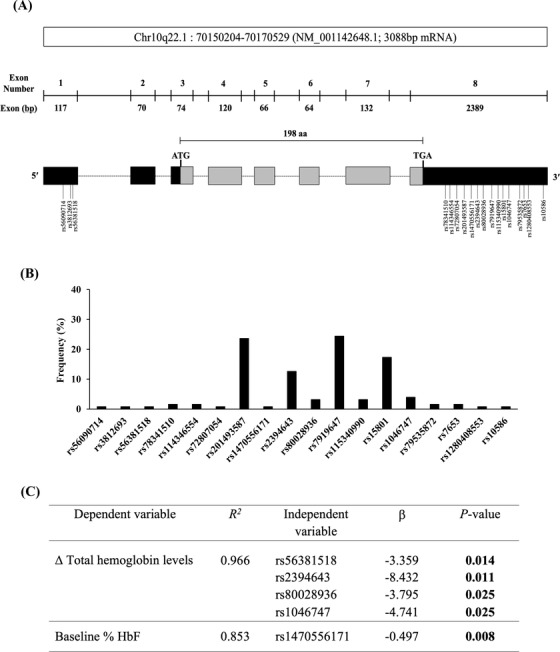
*SAR1A* coding‐region single nucleotide polymorphisms (SNPs) in adults with sickle cell disease (SCD) undergoing HU treatment. DNA samples from 32 adults with SCD undergoing HU treatment were analyzed for the presence of SNPs in the *SAR1A* coding regions and the association of *SAR1A* coding‐region SNPs with hematologic laboratory values. (A) Schematic overview of the *SAR1A* gene, which is located on chromosome 10q22.1 and contains 8 exons. Exon sizes are in base pairs (bp). Exons are shown as boxes. Protein‐coding regions are shown in gray, and UTRs are shown in black. DNA‐sequencing analysis for the cohort revealed 18 SNPs in *SAR1A* exons. (B) Frequency analysis of the occurrence of the 18 *SAR1A* exon SNPs in the cohort. (C) Multiple‐linear regression analysis of the 18 *SAR1A* exon SNPs in the cohort identified four SNPs significantly associated with a change in total hemoglobin levels after HU treatment (Δ total hemoglobin levels) with age adjustment and one SNP significantly associated with % HbF levels at baseline in the cohort. Δ Total hemoglobin levels is the difference in the concentration of total hemoglobin (g/dL) measured at the end of the study (following HU treatment) and the concentration measured prior to HU treatment in study participants. Baseline % HbF is the percent of HbF levels measured in study participants prior to HU treatment. *R* ^2^, coefficient of determination; *β*, coefficient of regression

Next, we analyzed these 18 *SAR1A* exon SNPs for association with hematological laboratory values determined in our cohort prior to and following HU treatment. We limited our statistical analysis to the 28 cohort members with an HbSS genotype (Table S1). Prior to multiple‐linear regression analyses in our cohort, we evaluated the correlation between age, gender, HU dose, or HU duration and hematologic laboratory values. Only age was associated with a change in total hemoglobin levels (*p* = 0.003) and with a change in absolute HbF levels (*p* = 0.009) after HU treatment. We therefore did not pursue using age, gender, HU dose, or HU duration status in our model, except we added age when analyzing change in total hemoglobin levels and in absolute HbF levels following HU treatment. The rs56381518, rs2394643, rs80028936, and rs1046747 SNPs were significantly associated with change in total hemoglobin levels after HU treatment by multiple‐linear regression analysis adjusted for age (Figure 1C). No significant associations with any of the 18 *SAR1A* exon SNPs were observed for changes in % HbF levels, absolute HbF levels, % F‐cells, or % F‐reticulocytes after HU treatment (data not shown). Consistently, Gardner et al. also reported that total hemoglobin levels were influenced by HbF‐boosting alleles in British SCD patients [15]. Interestingly, we found that one SNP (rs1470556171) was significantly associated with % HbF levels in the cohort measured prior to HU treatment (Figure 1C). In contrast, Pule et al. demonstrated that four selected SNPs in the *SAR1A* gene promoter (rs2310991, rs4282891, rs76901216, and rs76901220) were not associated with baseline % HbF levels in SCD patients [16].

In addition, when we used MicroSNiPer to examine the 15 SNPs that we identified in exon 8, all of which occurred in the 3′ UTR of *SAR1A*, numerous potential miRNA binding sites were identified (Table 1). The rs10586, rs7653, rs1046747, and rs7919647 SNPs were predicted targets for miRNAs that previously had only been identified in SCD patients with a mild phenotype (i.e., miR‐625‐5p, miR‐5003‐3p, miR‐1236‐5p, miR‐4271, miR‐345‐3p, miR‐4725‐3p, miR‐378a‐3p, miR‐548q, and miR‐135a‐3p) [13]. The rs10586 SNP was also identified as a potential target binding site for miR‐1200 and miR‐19b‐1‐5p, two miRNAs that have been reported to be differentially expressed in high HbF‐level conditions [12]. Furthermore, the rs2394643 SNP was found to be the potential binding site for miR‐3074‐3p, which was recently shown to be overexpressed in SCD patients undergoing HU treatment [17]. Further functional studies of miRNAs will be needed to better understand their influence on HU effects on γ‐globin expression in order to improve HU therapeutic approaches for SCD.

**TABLE 1:** List of potential miRNAs binding to single nucleotide polymorphisms (SNPs) identified in the 3′ untranslated region (UTR) of SAR1A

SNPs	miRNAs
rs10586	miR‐5003‐3p, miR‐345‐3p, miR‐1200, miR‐19b‐1‐5p, miR‐425‐5p, miR‐4728‐3p
rs7653	miR‐4668‐5p, miR‐3613‐3p, miR‐625‐5p, miR‐3148, miR‐4716‐3p, miR‐1236‐5p, miR‐4271, miR‐4725‐3p
rs79535872	miR‐3658, miR‐3613‐3p, miR‐1323
rs1046747	miR‐3646, miR‐3662, miR‐4306, miR‐378a‐3p, miR‐378c
rs15801	miR‐4667‐3p, miR‐4469, miR‐6511a‐3p, miR‐1281, miR‐205‐5p
rs115340990	miR‐1270, miR‐4784, miR‐3171, miR‐1206, miR‐1273f, miR‐620, miR‐143‐3p
rs7919647	miR‐548q, miR‐1250, miR‐4746‐3p, miR‐4299, miR‐135a‐3p, miR‐138‐5p, miR‐3692‐5p
rs2394643	miR‐616‐5p, miR‐3671, miR‐371b‐5p, miR‐3074‐3p, miR‐4698
rs72807054	miR‐1277‐5p, miR‐6082, miR‐374c‐5p

Our cohort study is limited by a relatively small sample size, and the results should therefore be viewed as preliminary. Despite this limitation, our findings show that individual *SAR1A*‐coding region SNPs are associated with a change in total hemoglobin level following treatment with HU, and with baseline % HbF levels in adults with SCD. We have illustrated the possible mechanisms of γ‐globin regulation through HU‐induced *SAR1A*, as well as through other HbF regulators and miRNAs, in Figure S1. Our data may prove useful in helping to predict variable HU responses in adults with SCD, as well as potential binding sites for miRNAs, both of which could be leveraged in therapeutics development.

## AUTHOR CONTRIBUTIONS

C.K. designed the research, performed the experiments, analyzed the results, and wrote the manuscript. C.K performed the experiments. G.K., J.Z., and W.A. contributed to the design of the study and scientific discussion of the results. J.G.T. evaluated the results and wrote the manuscript. G.P.R. designed the research, evaluated the results, wrote the manuscript, and gave final approval of the manuscript.

## CONFLICT OF INTEREST

The authors declare they have no conflicts of interest.

## ETHICS STATEMENT

This study has not been previously published elsewhere, properly credits the meaningful contributions of co‐authors and all sources used are properly disclosed.

## Supporting information

Supporting Information

## Data Availability

The data that support the findings of this study are available from the corresponding author upon reasonable request.
