# Supplementary material for: Single nucleotide polymorphisms in SAR1A coding regions in sickle cell disease and their potential miRNA binding sites
Source: EJHaem. 2022 Aug 3;3(4):1438–41. doi: 10.1002/jha2.542 (PMC9713051; doi:10.1002/jha2.542)
Supplement: Supplementary file 1 — Supporting Information [file JHA2-3-1438-s001.docx]

**Supporting information**

**Appendix S1. Supplemental Methods**

**Participants**

DNA samples and laboratory data were obtained from unrelated individuals with SCD who enrolled in a Sickle Cell Pulmonary Hypertension Screening Study at the National Institutes of Health and Howard University (ClinicalTrials.gov Identifier: NCT00011648). The study had enrolled 282 participants as of December, 2005, of which 269 had sufficient clinical data for inclusion criteria [1]. All participants were at least 18 years of age and provided written informed consent for participation in this Institutional Review Board–approved protocol. This cohort study included only the 32 of 269 participants who had quantitative high-performance liquid chromatography HbF measurements and other laboratory values determined before and during 2 years of HU therapy. Our cohort study consisted of 28 HbSS individuals, 2 HbSβ^+^-thalassemia individuals, 1 HbSβ^0^-thalassemia individual, and 1 HbSC individual. The mean age of our study participants was 37.3 years old (standard deviation [SD] = 11.3), and the gender composition of our cohort was 17 females and 15 males (demographic and hemoglobin genotype data for individual participants are presented in Table S1). As previously described, we used hospital and pharmacy records to determine the prescribed dose for HU treatment in each of our study participants [2]. The mean daily dose of HU was 16.87 mg/kg (SD = 5.60) (minimum: 8.56 mg/kg; maximum: 34.77 mg/kg). The mean duration of HU treatment was 21.4 months (SD = 8.09) (minimum: 8 months; maximum: 33 months).

**DNA sequencing**

Primers for amplification and sequencing were designed using Primer3 software (https://primer3.ut.ee) and the GenScript DNA sequencing primers design tool (https://www.genscript.com/tools/dna-sequencing-primer-design) (Table S2). Polymerase chain reaction (PCR) was performed using Platinum™ SuperFi™ PCR master mix (Thermo Fisher Scientific, Waltham, MA). The PCR conditions used were an initial denaturation at 98°C for 30 s, followed by 35 cycles at 98°C for 10 s, 58°C for 10 s, and 72°C for 30 s. Finally, an additional elongation step was carried out at 72°C for 5 min. The PCR products were purified using the QIAquick PCR purification kit (QIAGEN, Germantown, MD). Purified PCR products were directly sequenced in both directions by Eurofins Genomics (Louisville, KY). The SerialCloner (https://serial-cloner.en.softonic.com) and Clustal W (https://www.ebi.ac.uk/Tools/msa/clustalw2) programs were used to multiple align individual sequences with the human *SAR1A* reference sequence (GenBank accession number: NM_001142648.1 or December, 2013 assembly hg38: chr10q22.1:70150204-70170529).

**miRNA binding analysis**

Predicted miRNA binding to SNPs was identified using a web-based application, MicroSNiPer, which predicts the impact of an SNP on putative miRNA targets [3].

**Statistical analysis**

Multiple-linear regression (JMP 16.1.0) (JMP, Cary, NC) was used to determine the association between individual *SAR1A* SNPs and hematological laboratory data in study participants. *P* < 0.05 was considered as statistically significant. Only the 28 HbSS of 32 participants were included for our multiple-linear regression analyses model. Our analysis excluded age, gender, HU dose, and HU duration status from the model, except for when we analyzed the change in total hemoglobin levels and in absolute HbF levels following HU treatment, when we added age.

**References**

1. Taylor JGt, Ackah D, Cobb C, Orr N, Percy MJ, Sachdev V, et al. Mutations and polymorphisms in hemoglobin genes and the risk of pulmonary hypertension and death in sickle cell disease. Am J Hematol. 2008;83(1):6-14.

2. Fitzhugh CD, Hsieh MM, Allen D, Coles WA, Seamon C, Ring M, et al. Hydroxyurea-Increased Fetal Hemoglobin Is Associated with Less Organ Damage and Longer Survival in Adults with Sickle Cell Anemia. PLoS One. 2015;10(11):e0141706.

3. Barenboim M, Zoltick BJ, Guo Y, Weinberger DR. MicroSNiPer: a web tool for prediction of SNP effects on putative microRNA targets. Hum Mutat. 2010;31(11):1223-32.

4. Tang DC, Zhu J, Liu W, Chin K, Sun J, Chen L, et al. The hydroxyurea-induced small GTP-binding protein SAR modulates gamma-globin gene expression in human erythroid cells. Blood. 2005;106(9):3256-63.

5. Zhu J, Chin K, Aerbajinai W, Kumkhaek C, Li H, Rodgers GP. Hydroxyurea-inducible SAR1 gene acts through the Gialpha/JNK/Jun pathway to regulate gamma-globin expression. Blood. 2014;124(7):1146-56.

6. Mnika K, Mazandu GK, Jonas M, Pule GD, Chimusa ER, Hanchard NA, et al. Hydroxyurea-Induced miRNA Expression in Sickle Cell Disease Patients in Africa. Front Genet. 2019;10:509.

7. Pule GD, Mowla S, Novitzky N, Wonkam A. Hydroxyurea down-regulates BCL11A, KLF-1 and MYB through miRNA-mediated actions to induce gamma-globin expression: implications for new therapeutic approaches of sickle cell disease. Clin Transl Med. 2016;5(1):15.

**Table S1. Age, gender and hemoglobin genotype of study participants (n=32)**

| **Sample Number** | **Age** | **Gender** | **Hemoglobin Genotype** |
| --- | --- | --- | --- |
| 1 | 39 | F | SS |
| 2 | 33 | M | SS |
| 3 | 44 | F | SS |
| 4 | 47 | F | SS |
| 5 | 49 | M | SS |
| 6 | 51 | F | SS |
| 7 | 36 | F | SS |
| 8 | 44 | F | SS |
| 9 | 25 | F | SS |
| 10 | 23 | M | Sβ^0^-thalassemia (β^0^: IVS2-849 A/G) |
| 11 | 37 | M | SS |
| 12 | 38 | M | SS |
| 13 | 37 | M | SS |
| 14 | 43 | F | SS |
| 15 | 18 | M | SS |
| 16 | 30 | M | SS |
| 17 | 47 | M | SS |
| 18 | 25 | M | SS |
| 19 | 34 | F | SS |
| 20 | 22 | M | SS |
| 21 | 42 | F | SS |
| 22 | 27 | F | SS |
| 23 | 28 | F | SS |
| 24 | 58 | M | SC |
| 25 | 57 | F | Sβ^+^-thalassemia (β^+^:IVS2-848 C/A) |
| 26 | 51 | F | SS |
| 27 | 22 | M | SS |
| 28 | 52 | F | SS |
| 29 | 21 | M | Sβ^+^-thalassemia (β^+^:IVS1-5 G/A) |
| 30 | 27 | M | SS |
| 31 | 39 | F | SS |
| 32 | 47 | F | SS |

**Table S2.** List of primers used in PCR

| ***SAR1A* exon** | **Forward (5′ 🡪 3′)** | **Reverse (5′ 🡪 3′)** | **Product size (bp)** |
| --- | --- | --- | --- |
| 1 | CTGACTTTCCTGGTGCGTCT | GAGCGGAATGCAAGTGGAC | 634 |
| 2 | TTGCTTTCAAGGGAGAGACC | GGAGGGGAGGAAGCAAAGTA | 526 |
| 3 & 4 | CTTGGCTTTCCCAAATGCT | GTTGGCTTTTGGGACTTCAG | 603 |
| 5 | CTGGGCTGGTCTCAAAAATC | TTGAAGGGGACTTCTGTGTATT | 490 |
| 6 | CATGGACCATCAGTTTGCAT | CATTTTCCTCCTGCAACTAGG | 430 |
| 7 | TGTCATGGGTGTTATGTAATGTCA | CCTGATGAATGAATCCCATTT | 513 |
| 8 | GGCCTTGACCACATTTCTGT | TCCACAGCATGTTCCAAATC | 2748 |

**HU**

**miR-26b**

**miR-105-5p**

**miRNAs?**

**SAR1A**

**MYB**

**KLF-1**

**GATA-2**

**Giα/JNK/Jun**

**miR-148-3p**

**miR-32-5p**

**miR-340-5p**

**miR29c-3p**

**BCL11A**

**γ-globin**

**5′**

**3′**

**ε**

**Aγ**

**Gγ**

**δ**

**βε**

**LCR**

**Figure S1.** Schematic diagram of the possible mechanisms by which HU-induced *SAR1A* and other regulators mediate γ-globin expression. HU-induced SAR1A activates γ-globin expression through the induction of GATA-2 and the Giα/JNK/Jun pathway [4, 5]. miRNAs could indirectly regulate γ-globin expression through the critical regulators BCL11A [6], MYB [6, 7], or KLF-1, or through SAR1A. Figure adapted from Tang *et al*. and Pule *et al*. [4, 7].
